# Operation for Recurring Dislocation of the Lower Jaw

**Published:** 1893-03

**Authors:** 


AMERICAN JOURNAL

—OF—

DENTAL SCIENCE.

Vol. XXVI. Baltimore, March, 1893. No. 11.

ARTICLE I.

### OPERATION FOR RECURRING DISLOCATION OF THE LOWER JAW.

In the *British Medical Journal*, Dr. Marsh, of Birmingham writes:

Mrs. A. O., aged 23, a stout, healthy-looking woman, was admitted into Queen's Hospital under my care on May 13, 1890. Five months previously, and a fortnight after confinement, she bilaterally dislocated her lower jaw when gaping. It was reduced, but in a fortnight's time was again dislocated by gaping. Since then recurrence has been frequent, some days happening many times—in fact, almost always if the mouth is incautiously opened. The dislocation is always easily reduced by her usual medical attendant, but if she is away from home difficulty is sometimes experienced, and the manipulation causes her considerable suffering. Restraining bandages had been worn, but the patient objected to them as a permanence, and was extremely anxious to have something done and was willing to submit to any operation offering even a chance of relief.

On May 15th, under anæsthesia the left temporo-maxillary articulation was exposed by an incision about an inch long, extending downwards from the zygoma, half an inch in front of the auricle. The capsule was opened and the interarticular fibrocartilage was found to be exceedingly loosely attached. The external lateral ligament could be clearly defined. Following Professor Annandale's method of fixing the cartilage in cases of subluxation, a catgut suture was passed through the periosteal attachment of the capsule to the zygoma and through the margin of the cartilage; but in tying it in the deep and narrow wound it cut through the cantilage; fine silver wire was substituted, twisted and cut off short. A second suture was now passed through the external lateral ligament and through the periosteal attachment of the capsule as far posteriorly as possible, so as to make a fold or tuck in the ligament and bind it down to the adjoining structures. The wound was then closed. It was anticipated that these sutures, assisted by the adhesions which would form during the healing process between the exposed part of the capsule and the surrounding tissues, would fulfill the first two aims of the method, and that careful asepsis would ensure the third.

Primary union took place, and in twelve days the patient returned home, wearing an elastic bandage to restrain movement, and for a time being limited to slop diet. She wore the bandage continuously for two months, and during this time there was no recurrence on either side. When she ceased to wear it dislocation of the right condyle soon took place, and became of frequent occurrence; the left remained in place in spite of extra strain thrown upon it during the manipulation for reducing the opposite side. The right articulation was therefore operated upon in a somewhat similar manner on September 25th, 1890, but only one silk suture was used; this was passed through the external lateral ligament, the periosteal attachment of this capsule on the outer side of the glenoid margin, the interarticular fibrocartilage, back through the external lateral ligament, and then tied. Primary union again took place, except along a small drainage tube track, and the patient went home on October 8th, observing the same precautions as on the previous occasion.

Since the second operation there has been no recurrence of dislocation, and no trouble whatever has been experienced from the buried sutures. The cicatrices are hardly visible even on close inspection; there is a distance of 1½ in. between the front incisor teeth when the mouth is open, and when the jaws are closed the teeth meet accurately. She can open her mouth widely or gape without fear, and can eat anything; she is, in fact, *re* the dislocation, perfectly well.

The operation, though apparently simple, is not a very easy one to perform. The wound must be limited in length because of the anatomical surroundings; the tissues cut through are very vascular, and it is difficult both to pass the suture, and to tie it sufficiently tight without cutting through the fibrocartilage. On this latter account aseptic silk or tendon will probably be found the most suitable material for the suture. In view of the possibility of suppurative arthritis and subsequent ankylosis, it is advisable to operate on one side at a time. The second operation should preferably take place soon after the healing of the first wound, and before the elastic bandage is discarded.

A sufficient length of time has now elapsed—over eighteen months since the second operation—to establish beyond doubt the permanent character of the result obtained by this method of treatment, the success of which has in this instance been so complete as to justify its employment in other similar cases.

